# Outcome of Scaphoid Nonunion Using Open Reduction and Internal Fixation With Iliac Crest Bone Graft (Fisk-Fernandez Technique)

**DOI:** 10.7759/cureus.34661

**Published:** 2023-02-05

**Authors:** Swagat Mahapatra, Pankaj Aggarwal, Prakhar Mishra, Sachin Avasthi, Jitesh Arora, Satyam Singh, Mohd A Aslam

**Affiliations:** 1 Department of Orthopedic Surgery, Dr. Ram Manohar Lohia Institute of Medical Sciences, Lucknow, IND

**Keywords:** bone graft, fisk-fernandez, iliac crest, non-union, scaphoid

## Abstract

Introduction

The scaphoid is the most common carpal bone to be fractured and has a high propensity for nonunion. Restoration of scaphoid length mitigates the chances of long-term complications. The aim of this study was to assess the functional outcome of the Fisk-Fernandez technique for the treatment of scaphoid nonunion by using open reduction and internal fixation with trapezoidal iliac crest bone graft.

Materials and methods

Fisk-Fernandez technique was used to manage scaphoid nonunion in 31 patients at a tertiary care hospital with follow-up at six weeks, 12 weeks, and 24 weeks. An objective assessment of the outcome was done using a comparison of the pre- and postoperative scaphoid score, QuickDASH, and visual analog score.

Discussion

The scaphoid is one of the most common carpal bones to get fractured. Anatomical factors, late presentation, and delay in diagnosis render it to usually land in nonunion. A comparison of the preoperative scaphoid, QuickDASH, and VAS scores with six-week, 12-week, and 24-week postoperative scores was made and was found to be statistically significant (p<0.001). Ninety-three percent of patients subjectively reported satisfaction after treatment. Though revascularization was not assessed, the bony union was observed in all the patients.

Conclusion

The operative technique proposed by Fisk-Fernandez is effective in correcting deformity of the scaphoid as well as providing satisfactory functional outcomes in patients with scaphoid nonunion.

## Introduction

A scaphoid fracture is the most common bone to be fractured among the carpal bones accounting for 60% of all cases [1]. The scaphoid is the linked bone between the proximal and distal rows of the carpals and plays a vital role in carpal stability [2]. Nonunion rates in scaphoids are affected by fracture location, displacement, instability, and time to treatment. Tenderness at the anatomical snuff box is the most important sign of scaphoid nonunion [3]. Approximately 10% of scaphoid fracture ends up in nonunion. Typically the scaphoid flexes with wrist flexion and radial deviation and extends during wrist extension and ulnar deviation, so nonunion of fracture scaphoid can lead to humpback (excessive flexion) deformity, SNAC (scaphoid nonunion advance collapse), carpal collapse, and degenerative wrist arthritis [4]. In established scaphoid nonunion with carpal instability, correction of the flexion deformity and restoration of normal scaphoid length re-establishes normal tension in the palmar radiocarpal ligaments, which in turn corrects the pathological rotation of lunate [5]. Most cases of scaphoid nonunion with a pattern of dorsal intercalated segmental instability of the carpus result in a palmar flexion deformity of the scaphoid that requires a palmar opening wedge grafting. Current evidence does not demonstrate a single superior method of its treatment, and different authors have used various methods. Our study aims to analyze the outcome of the Fisk-Fernandez procedure in scaphoid nonunions with flexion deformity of the scaphoid with carpal instability [5-7].

## Materials and methods

All patients with a scaphoid fracture nonunion at the scaphoid waist and intervened by the Fisk-Fernandez procedure from April 2017 to April 2021 were retrospectively included. The institutional ethical committee approved the study (IEC no. 17/22). All patients with fracture scaphoid nonunion were intervened by a nonvascularized iliac crest bone graft and followed for more than six months. A total of 31 patients fulfilled the inclusion criteria and were included in the study. Patients with nonunion of scaphoid fracture and who were skeletally mature and below 60 years of age without any related injuries in the ipsilateral upper limb and without any evidence of wrist arthritis were included in our study. Radiographs of posteroanterior view in 15-degree ulnar deviation, pronated oblique, supinated oblique, and lateral views were taken preoperatively.

All surgeries were performed under general anesthesia, and a tourniquet was used in the arm. A volar approach to the scaphoid was used; the nonunion site was exposed and confirmed under C-arm image guidance. The nonunion site was freshened and distracted with the help of two mini k-wires in the proximal and distal poles, respectively. After measuring the amount of graft required, a graft of equal dimension was harvested from the iliac crest. The graft was impacted into the nonunion site and held provisionally with k wires, followed by definitive fixation with a headless compression screw under C arm image guidance.

**Figure 1:**
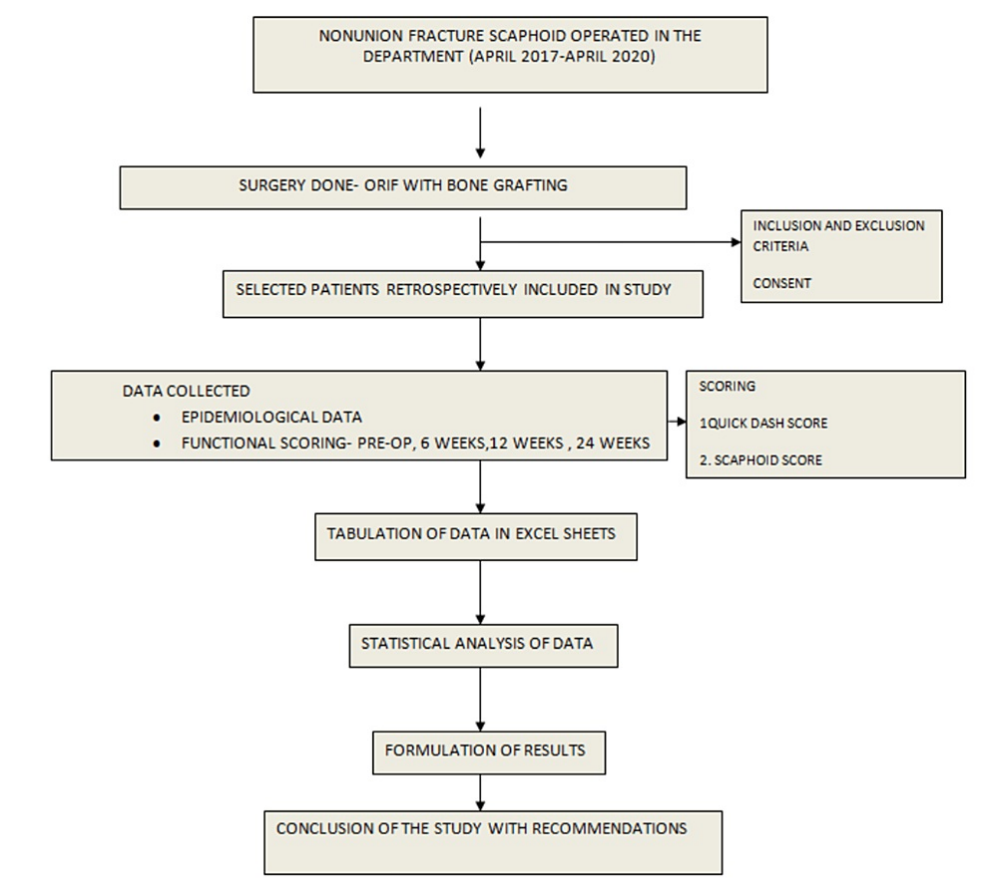
Methodology flowchart

Scapholunate angle and scaphoid length were measured in the lateral X-rays both pre- and postoperatively, along with the QuickDASH score and visual analog scale (VAS). A single surgeon performed all surgeries. After surgery, all patients were given immobilization for four weeks and followed up at six, 12, and 24 weeks (Figure 1).

Data were compiled using MS EXCEL and analyzed with Statistical Package for Social Science, SPSS version 25 (IBM Corp, Armonk, NY). Categorical variables were expressed in frequency and percentage, and quantitative data were expressed in terms of mean and standard deviation. Paired t-test was used to test the difference in means. p-Value <0.05 was considered statistically significant.

## Results

**Figure 2:**
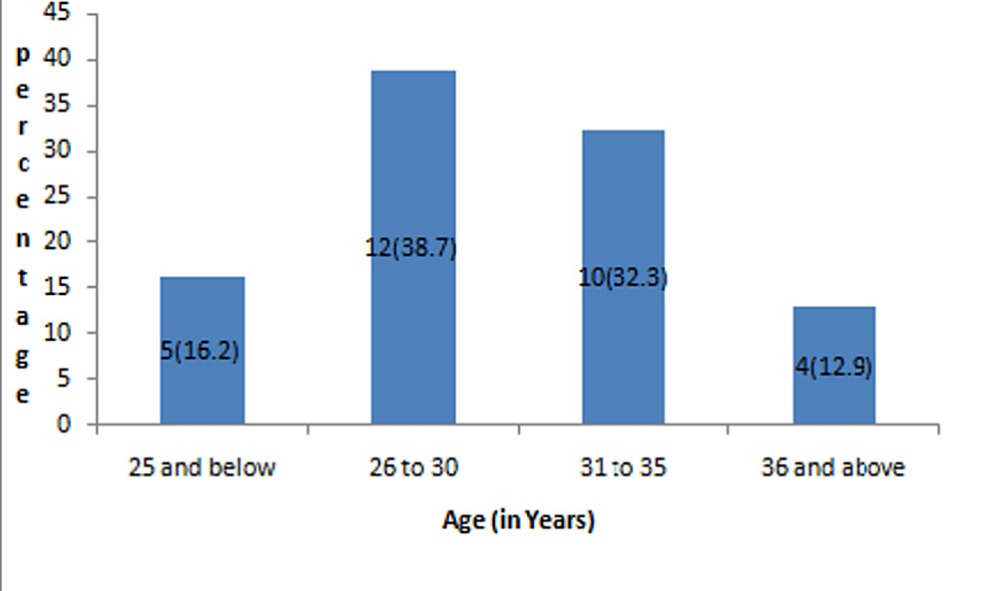
Distribution of participants on the basis of age

A total of 31 patients participated in the study. The mean age ± SD of participants in the study is 29.87 ± 4.7 years. The maximum age of the patient in our study was 40 years, and the minimum was 22 years. Out of 31 participants, the majority, 12 (38.7%), belonged to 26-30 years, followed by 10 (32.3%) belonging to 31-35 years age group, 5 (16.2%) and 4 (12.9%) belonging to <25 years and >36 years age group, respectively (Figure 2). 

**Figure 3:**
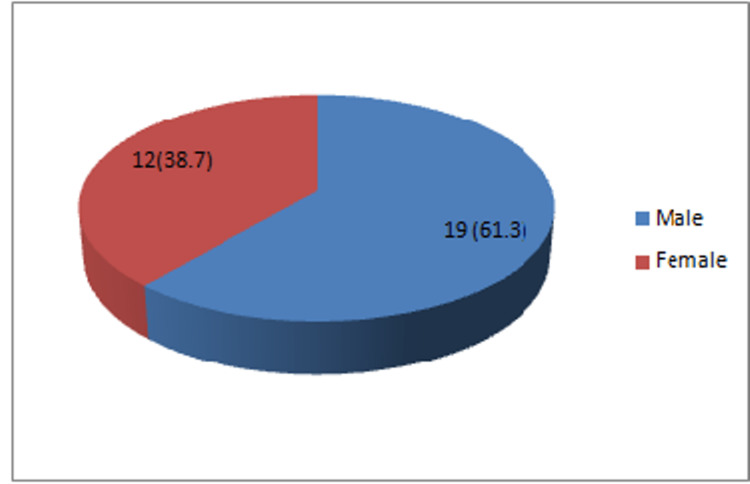
Distribution on the basis of gender

Out of 31 participants, 19 (61.3%) were males and 12 (38.7%) were females (Figure 3).

**Figure 4:**
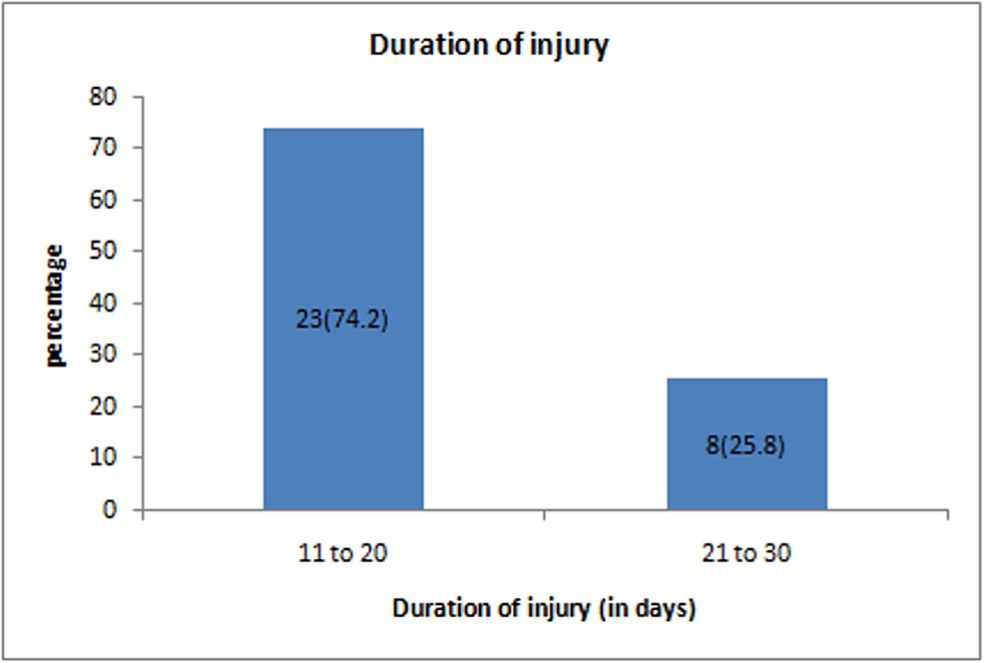
Distribution of study participants on the basis of duration of injury

The mean ± SD duration of the injury was 18.45 ± 4.62 days (range 11-29). Out of 31 participants, the majority, 23 (74.2%), had a duration of injury between 11 and 20 days (Figure 4). 

The mean ± SD scaphoid score of the preoperative group was 6 ± 0.00. During follow-ups, the mean scaphoid score showed a decreasing trend. At six weeks, the mean score was 4.71 ± 0.693, which came down to 2.32 ± 0.54 at 12 weeks and 24 weeks; it was 0.13 ± 0.34 (Table 1). The mean ± SD QuickDASH score in the preoperative group was 83.45 ± 6.61. During follow-ups, the mean QuickDASH score showed a decreasing trend. At six weeks, the mean ± SD score was 65.87 ± 4.15, which came down to 33.84 ± 5.67 at 12 weeks and 24 weeks it was 8.42 ± 2.08 (Table 1). 

**Table 1:** Distribution of mean, SD, and SE of scaphoid score and QuickDASH score preoperatively, and at six weeks, 12 weeks, and 24 weeks SD, standard deviation; SE, standard error.

Score	Preoperative	6 weeks	12 weeks	24 weeks
Scaphoid score				
Mean	6	4.71	2.32	0.13
SD	0	0.693	0.541	0.341
SE mean	0	0.124	0.097	0.061
QuickDASH score				
Mean	83.45	65.87	33.84	8.42
Standard deviation	6.61	4.15	5.67	2.08
Standard error mean	1.19	0.75	1.19	0.37

The mean ± SD VAS score in the preoperative group was 79.03 ± 10.23, which decreased to 7.71 ± 1.53 at the 24-week follow-up (Table 2). 

**Table 2:** Distribution of mean, SD, and SE of VAS score preoperatively and at 24 weeks SD, standard deviation; SE, standard error; VAS, visual analog scale.

VAS	Preoperative	24 weeks
Mean	79.03	7.71
SD	10.23	1.53
SE mean	1.84	0.28

**Figure 5:**
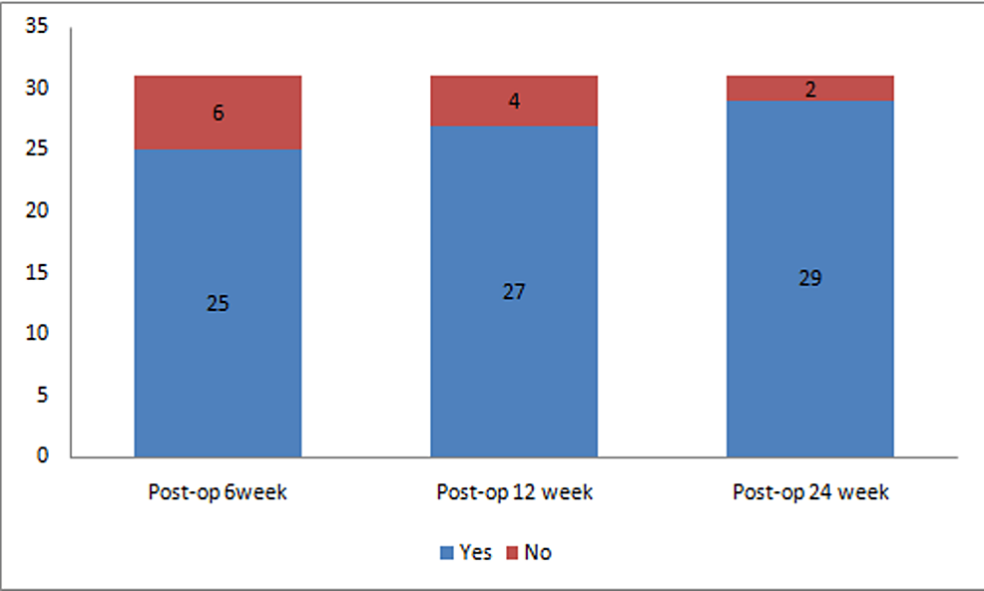
Subjective assessment of patient satisfaction

Subjective assessment of patient satisfaction was done on sequential visits on a personal interview basis questioning whether they got relief in their presenting complaints in the sixth, 12th, and 24th-week postoperative visit. Twenty-nine out of 31 (93%) patients reported symptomatic relief in their initial presenting complaints (Figure 5). 

The difference in the mean of the scaphoid preoperative score when compared with six weeks, 12 weeks, and 24 weeks was found to be statistically significant (p-value < 0.001) (Table 3). 

**Table 3:** Association of preoperative scaphoid score with six weeks', 12 weeks', and 24 weeks' scaphoid score

Scaphoid score	t-Value	Df	p-Value
Preop vs 6 weeks	10.37	30	<0.001
Preop vs 12 weeks	37.86	30	<0.001
Preop vs 24 weeks	95.92	30	<0.001

The difference in the mean of QuickDASH preoperative score when compared with six weeks, 12 weeks, and 24 weeks was found to be statistically significant (p-value < 0.001) (Table 4). 

**Table 4:** Association of preoperative QuickDASH score with six weeks', 12 weeks', and 24 weeks' QuickDASH score

QuickDASH score	t-Value	Df	p-Value
Preop vs 6 weeks	12.69	30	<0.001
Preop vs 12 weeks	32.26	30	<0.001
Preop vs 24 weeks	60.49	30	<0.001

The difference in the mean of the VAS score when compared with 24 weeks' VAS score was found to be statistically significant (p-value ≤ 0.001) (Table 5).

**Table 5:** Association of preoperative VAS score with 24 weeks' VAS score VAS, visual analog scale.

VAS	t-Value	Df	p-Value
Preop vs 24 weeks	37.39	30	<0.001

## Discussion

Scaphoid fracture accounts for more than 70% of carpal fractures and about 2% of total fractures [8]. The majority of these fractures can be managed conservatively with cast immobilization; however, some cases require surgical intervention [9]. Anatomical peculiarities like intraarticular position, lack of attachments, and precarious blood supply render scaphoid fractures prone to nonunion in approximately 10% of cases [4].

Clinically scaphoid nonunions may present with minimal symptoms like radial wrist pain, with radial sides swelling, range-of-motion restriction, tenderness in the anatomical snuff box, and decreased grip strength.

Scaphoid nonunion, if left untreated for a long duration, progresses further to wrist arthritis and impaired wrist joint function [10]. Both patient and fracture characteristics have to be considered while planning management. Common complications of nonunion of scaphoid fracture are malalignment and progressive wrist arthritis.

Numerous fixation methods and grafting options are mentioned in the literature to fix scaphoid fractures. The most commonly used autogenous grafts include cortico-cancellous or cancellous iliac grafts, cortico-cancellous and cancellous distal radius grafts, and grafts from radial styloid [11-14]. The osteogenic features of these grafts as well as the strength of the graft under physiologic stress determine osseointegration [15].

The technique proposed by Matti-Russe offered satisfactory long-term results of about 90% consolidation [16,17]. However, the demerits of the procedure included joint stiffness due to prolonged immobilization extending up to six months and inter-scaphoid angulation inadequacy.

Reduction of deformity by inter-positioning of anterior wedge graft enables consolidation in the correct position as postulated by Fisk et al. Fernandez modified this technique by the interposition of iliac crest graft and internal fixation. Similar results were proposed by Chen et al., Daly et al., and Finsen et al. [18-20]. 

In our study, the objective assessment was measured through the scaphoid score. The difference in the mean of the scaphoid preoperative score when compared with six weeks, 12 weeks, and 24 weeks was found to be statistically significant (p-value < 0.001). Similar results were shown in previous studies by Robbins et al. and Steinmann et al. [21,22].

The mean ± SD QuickDASH score preoperatively was 83.45 ± 6.61. During follow-ups, the mean QuickDASH score shows a decreasing trend. At six weeks, the mean ± SD score was 65.87 ± 4.15, which comes down to 33.84 ± 5.67 at 12 weeks and 24 weeks; it was 8.42 ± 2.08, which improved significantly in the postoperative period.

Subjective assessment of patient satisfaction was done by conducting a personal interview with each patient in which the patient reported postoperative relief in their chief complaints as “Yes/No”. Ninety-three percent of patients reported satisfactory relief in their presenting complaints at the 24th week postoperative visit.

Addressing scaphoid nonunions with cortico-cancellous iliac bone graft effectively reduces the humpback deformity by decreasing the scapholunate angle due to the wedge shape of the graft. Daly et al. in 1996 reported similar results [19].

Limitations of this study include its retrospective nature, intervention done by a single surgeon, and this study being conducted at a single tertiary center. Data collection was done by history and interview methods which includes recall bias.

## Conclusions

The scaphoid is the most commonly fractured carpal bone. Though the union rate varies between 55% and 100%, about 10% of scaphoid fractures land up in nonunion. Factors affecting nonunion are the location of the fracture, displacement, stability, and time for initiation of treatment. Despite many methods mentioned in the literature, there is currently no consensus on the best management possible. Early diagnosis and timely intervention by surgical techniques prevent the onset of arthritis in the wrist joint. The Fisk-Fernandez procedure provides acceptable restoration in scaphoid parameters like scaphoid length and correction of flexion deformity. Long-term results are recommendable and provide preservation of wrist function and early return to professional life.
